# Supplementary material for: Design, Synthesis and Molecular Docking Analysis of Flavonoid Derivatives as Potential Telomerase Inhibitors
Source: Molecules. 2019 Sep 1;24(17):3180. doi: 10.3390/molecules24173180 (PMC6749477; doi:10.3390/molecules24173180)
Supplement: Supplementary file 1 [file molecules-24-03180-s001.pdf]

Supporting Information

Design, Synthesis and Molecular Docking Analysis of Flavonoid Derivatives as Potential Telomerase Inhibitors

Zhan-Fang Fan,†,§ Sai-Tim Ho,‡,§ Rui Wen,† Ya Fu,† Lei Zhang,† Jian Wang,† Chun Hu,† Pang-Chui Shaw,‡,* Yang Liu,†,* Mao-Sheng Cheng†,*

†Key Laboratory of Structure-Based Drugs Design and Discovery (Ministry of Education), School of Pharmaceutical Engineering, Shenyang Pharmaceutical University, Shenyang 110016 China

‡School of Life Sciences, The Chinese University of Hong Kong, Shatin, Hong Kong SAR China

* Correspondence: pcshaw@cuhk.edu.hk (P.-C. S.); y.liu@syphu.edu.cn (Y. L.); mscheng@syphu.edu.cn (M.-S. C.)

§ Z.-F. F. and S.-T. H. contributed equally to this work.

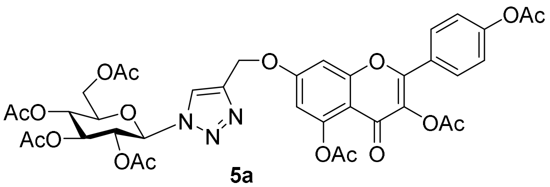

5a

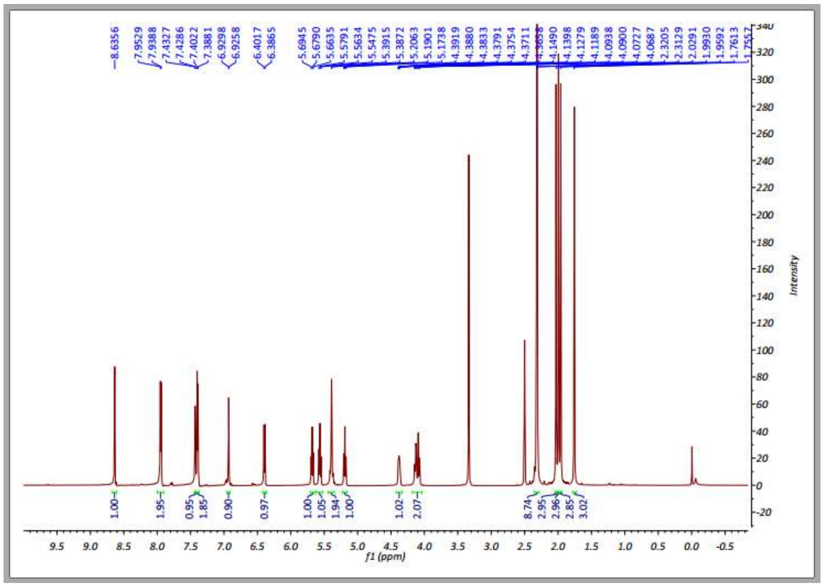

Figure S1. The ¹H-NMR of compound 5a

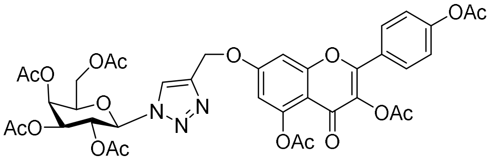

5b

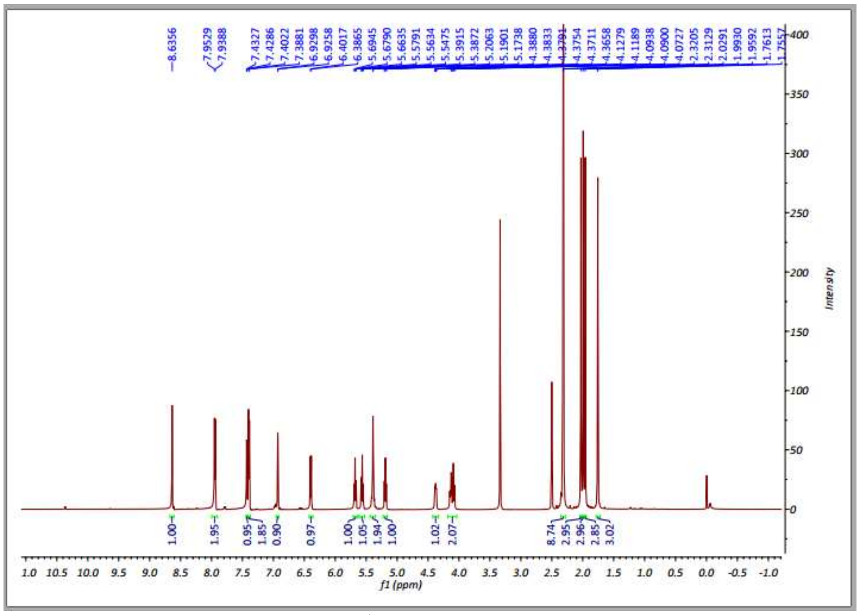

Figure S2. The ¹H-NMR of compound 5b

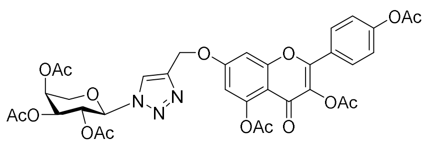

5c

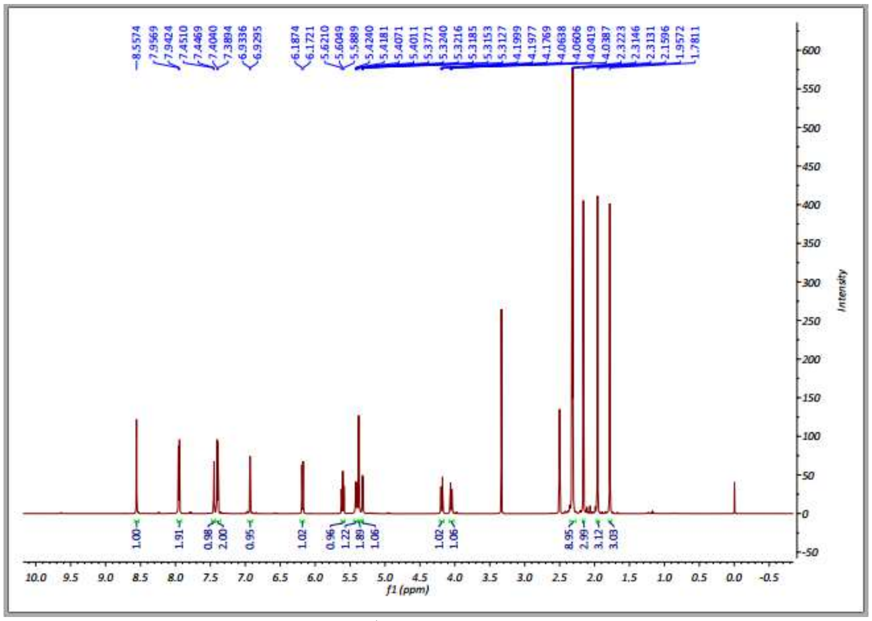

Figure S3. The ¹H-NMR of compound 5c

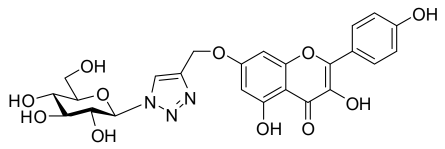

6a

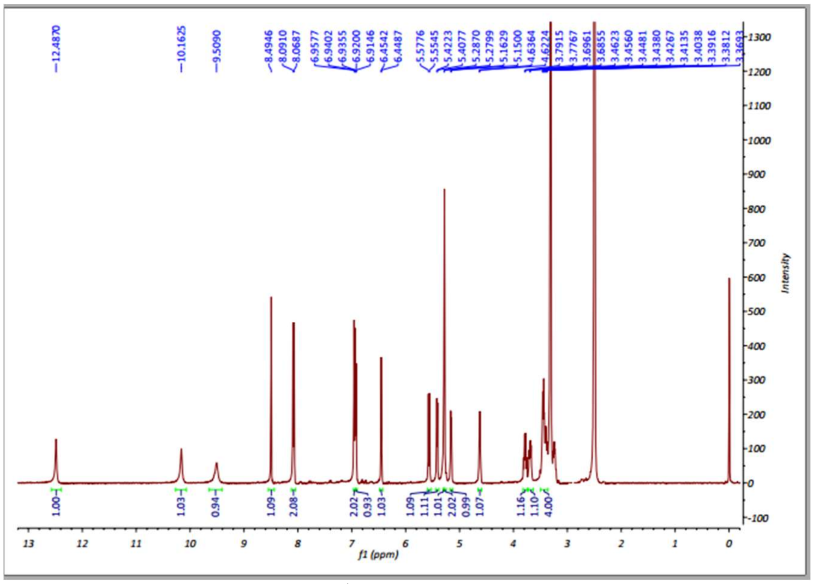

Figure S4. The ^{1}H-NMR of compound 6a

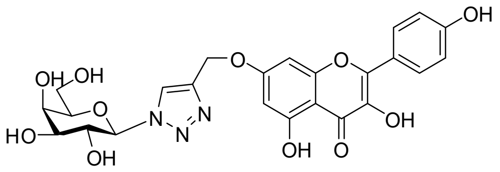

6b

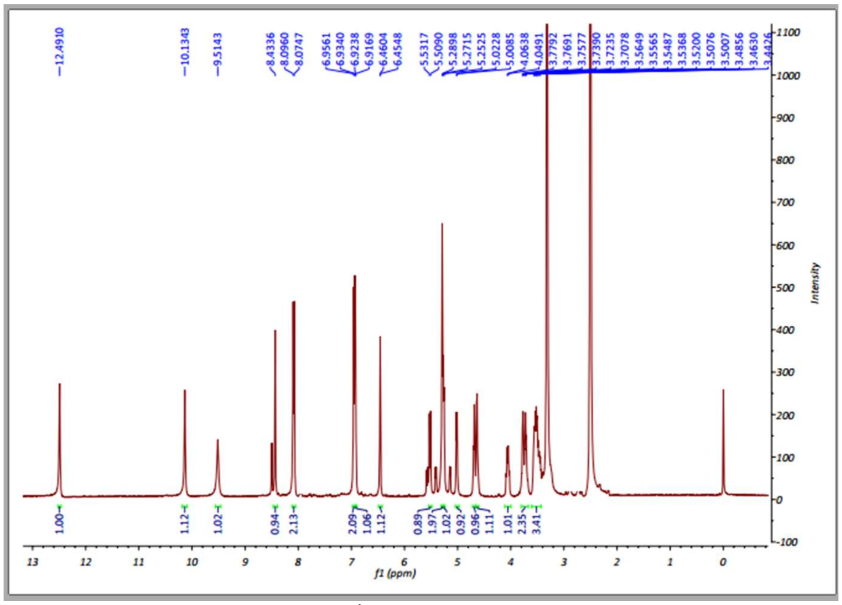

Figure S5. The ^{1}H-NMR of compound 6b

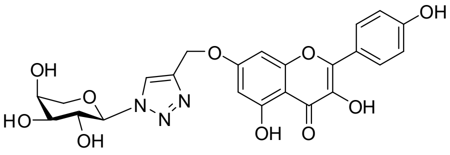

6c

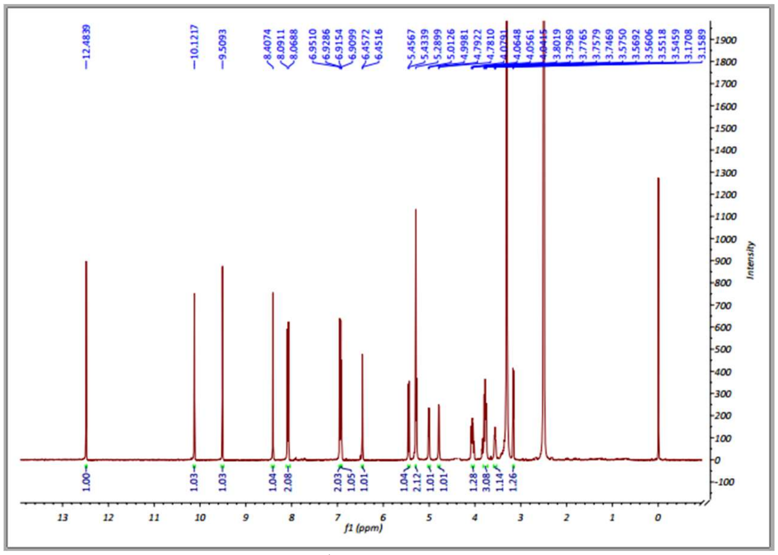

Figure S6. The ¹H-NMR of compound 6c

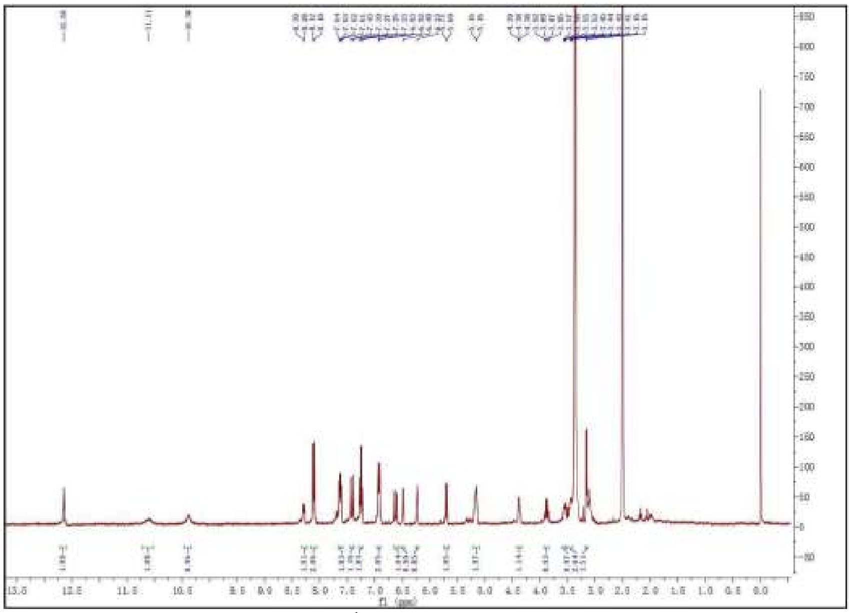

Figure S7. The ¹H-NMR of compound 14a

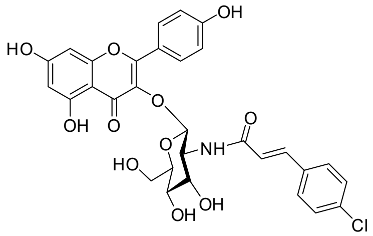

14b

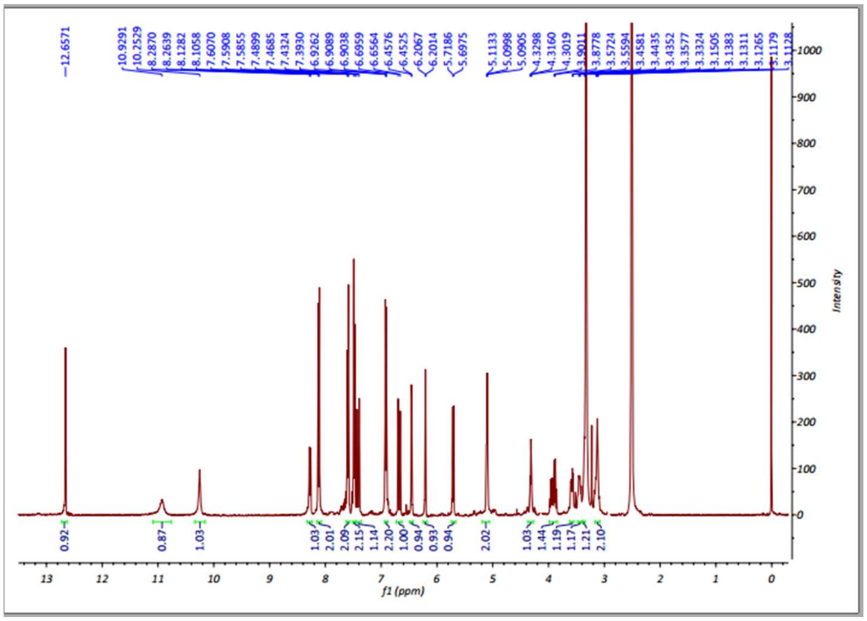

Figure S8. The ¹H-NMR of compound 14b

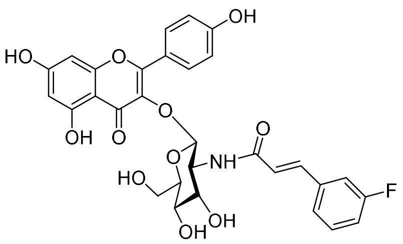

14c

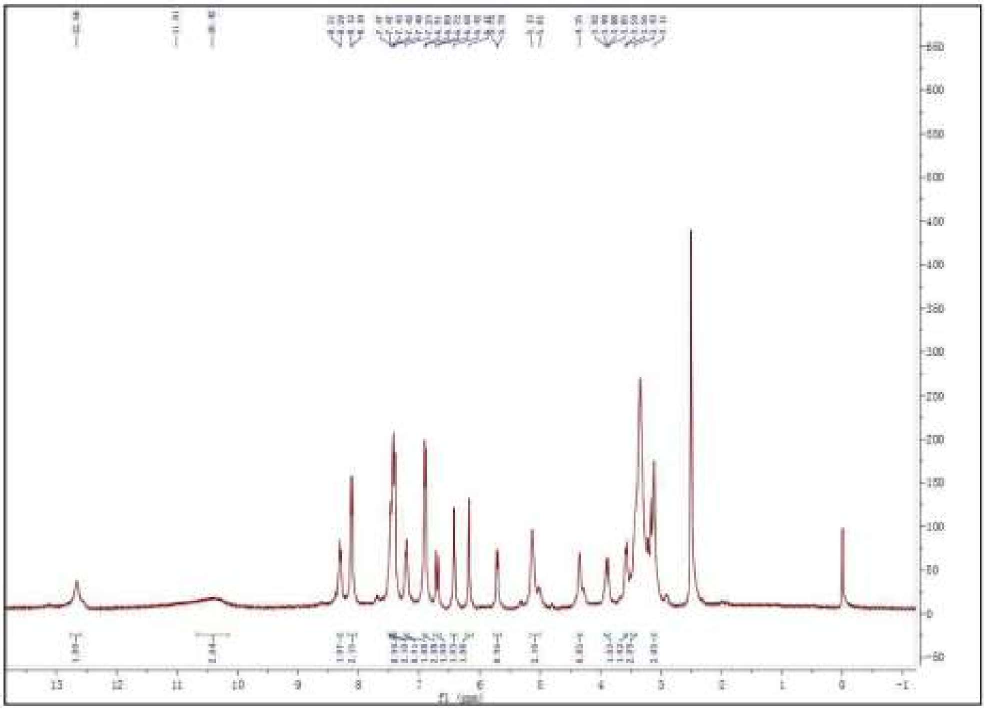

Figure S9. The ^{1}H-NMR of compound 14c

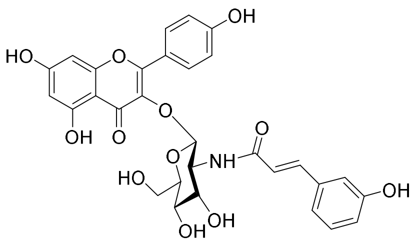

14d

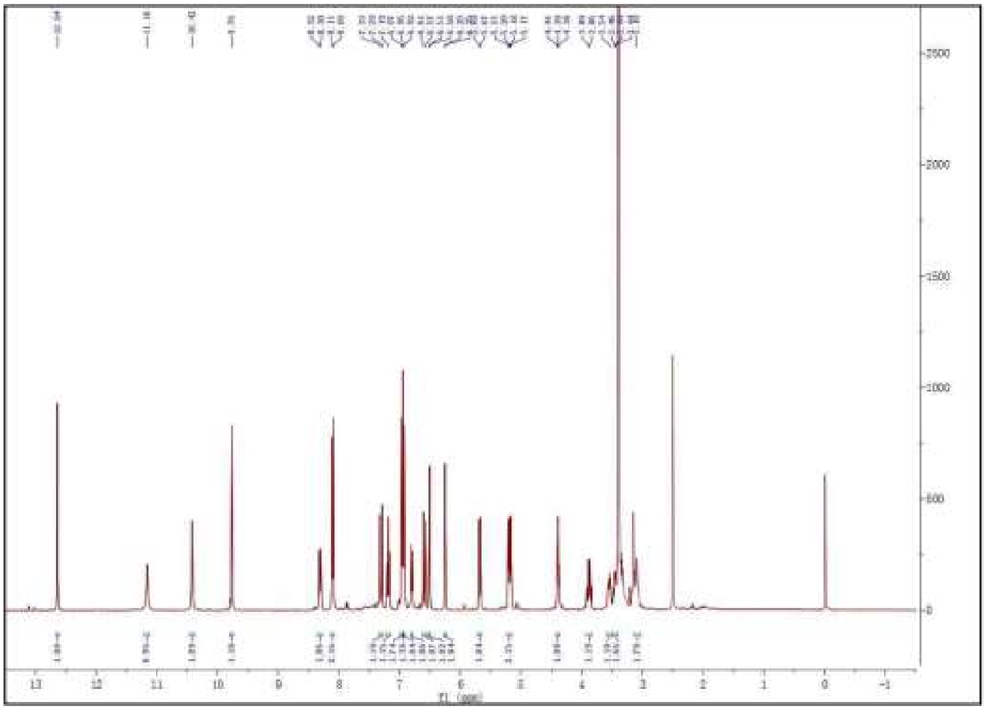

Figure S10. The ^{1}H-NMR of compound 14d

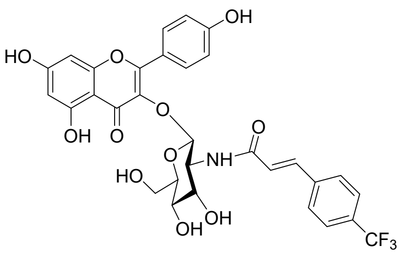

14e

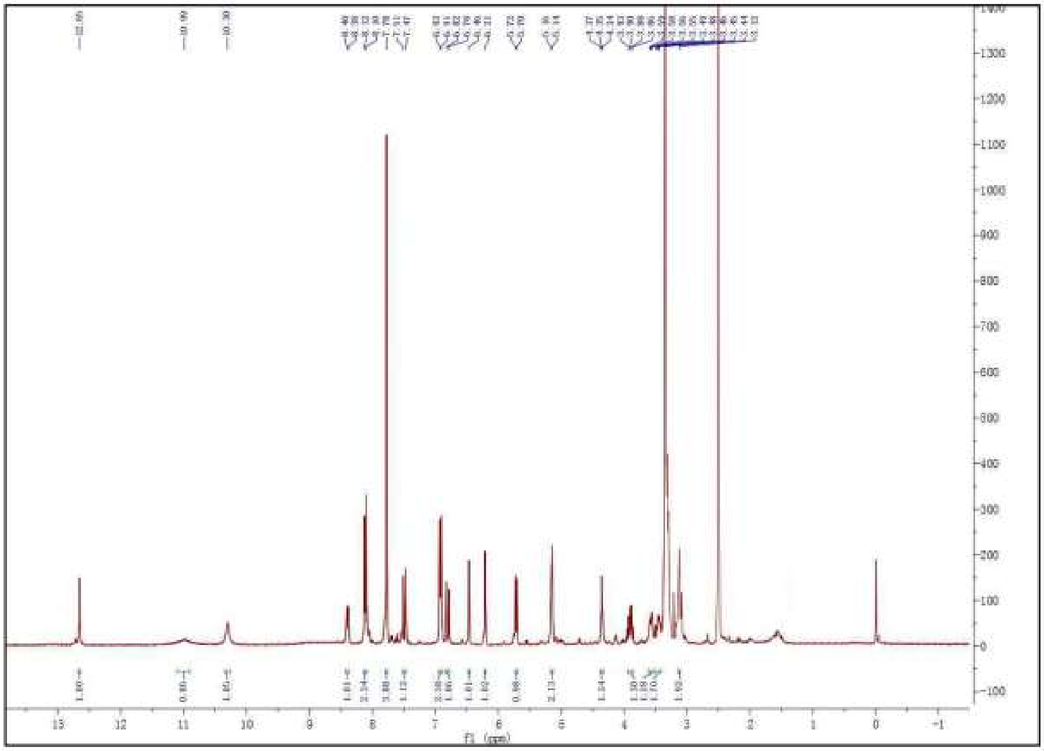

Figure S11. The ^{1}H-NMR of compound 14e

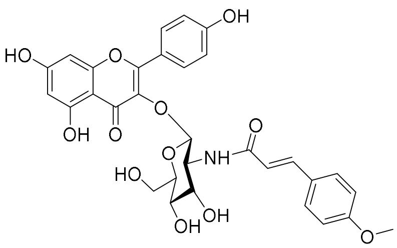

14f

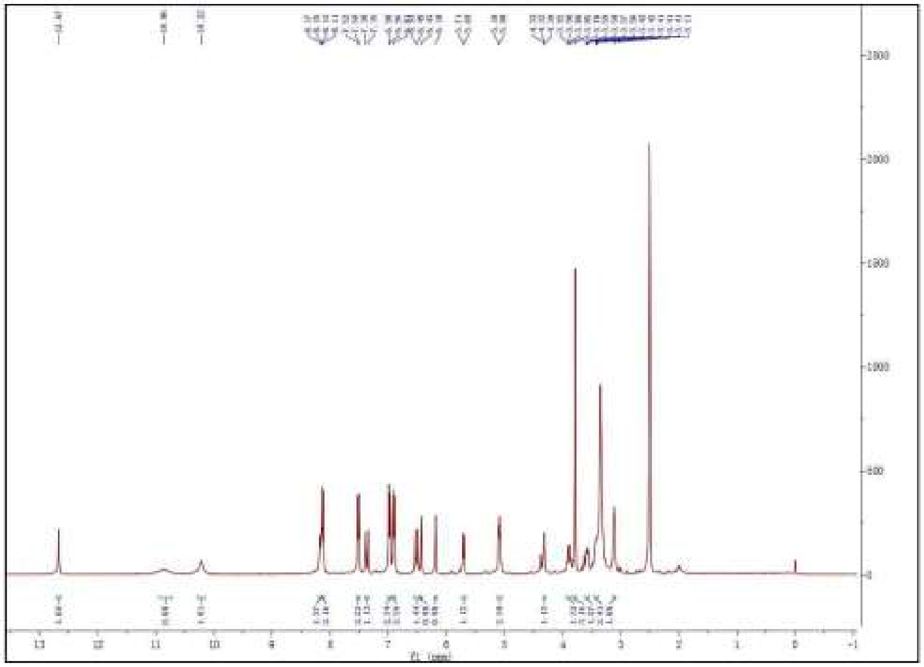

Figure S12. The ^{1}H-NMR of compound 14f
